# Isolated Renal Mucormycosis after Liver Transplantation:An Unusual Case Report

**Published:** 2012-07-30

**Authors:** B Geramizadeh, K Kazemi, A R Shamsaifar, A Bahraini, S Nikeghbalian, S A Malekhosseini

**Affiliations:** 1 Transplant Research Center, Shiraz University of Medical Sciences, Shiraz, Iran; 2 Transplant Ward, Department of Surgery, Shiraz University of Medical Sciences, Shiraz, Iran

**Keywords:** Kidney, Mucormycosis, Liver transplantation

## Abstract

Mucormycosis is a rare complication of immunosuppression. Most of the reported cases have been rhinocerebral or disseminated. Isolated renal involvement is extremely rare and until now less than 30 patients have been reported in the English literature. Isolated renal mucormycosis with renal artery rupture in a liver transplant patient has not been reported so far. Herein we report an extremely rare case of isolated renal mucormycosis in a liver transplant patient who was successfully treated with nephrectomy.

## Introduction

Infections are common complications after liver transplantation and they are considered as a huge threat to the patient's survival.[1] Fungal infections are particularly important among other infections because of special symptoms and high mortality rate.[2] Mucormycosis is a rare complication of solid organ transplantation associated with immunosuppressive therapy.[3] Renal mucormycosis as an isolated fungal infection has very rarely been reported in immunosuppressant patients and also there are few reported cases in patients with no known underlying disease.[4] To the best of our knowledge, this is the first case of renal infarction secondary to rupture of renal artery because of mucormycosis in a liver transplant patient.

## Case Report

A 40-year-old man, known case of cryptogenic cirrhosis, underwent liver transplantation from an ABO identical deceased donor. His posttransplantation course was uneventful and he left the hospital on good condition. He was receiving mycophenolate mofetil, cyclosporine and prednisolone. After 20 days, he developed fever and flank pain. Physical examination was unremarkable except for mild tenderness in left flank area. Laboratory investigation composed of blood and urine culture, complete blood count and liver function tests were normal.

**Figure 1:**
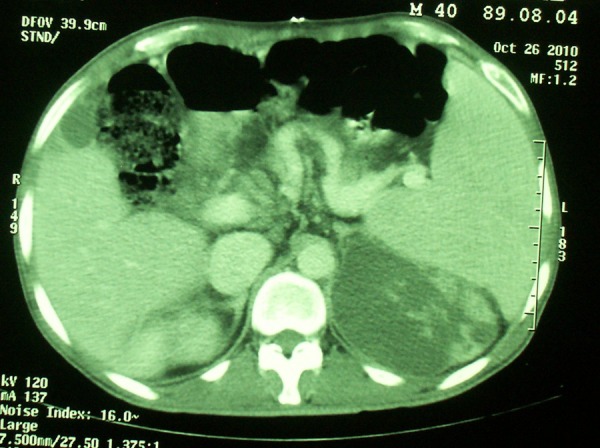
CT scan of the abdomen shows a cystic lesion in the kidney

Abdominal ultrasonography showed a 10X2 cm collection in left perirenal area. The fluid was aspirated and cultured. The smears of aspirated material showed nonseptated hyphea consistent with mucor. Antifungal therapy mainly composed of amphotericin B was started. However fever continued and further investigation by CT scan revealed cystic hypoattenuated lesion in the medial aspect of left kidney (Figure 1).

**Figure 2:**
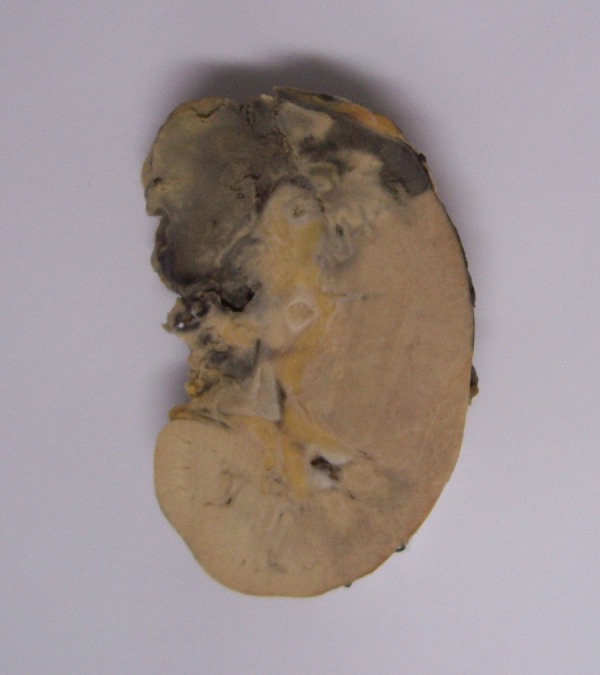
Gross of the kidney shows large area of infarction.

**Figure 3:**
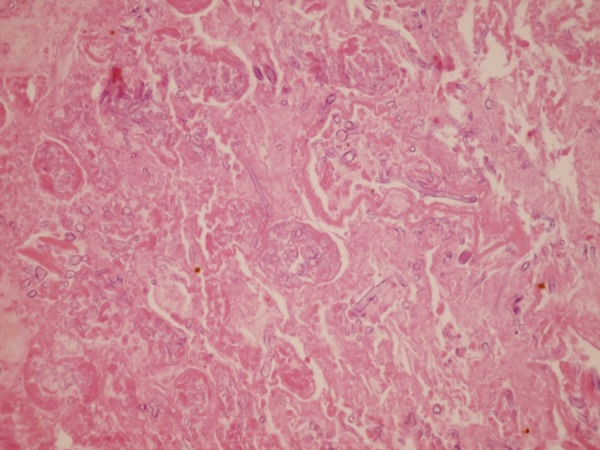
Microscopic sections of the kidney show coagulative necrosis containing many fungal elements. (H and E X 250)

X-ray of the lung and chest CT scan were unremarkable. Despite of fluid drainage by catheter, the fever continued, so decision was made to explore the patient in the operation room. Some necrotic and thick fluid was drained and the space was washed thoroughly. After all these efforts his fever continued and he developed abdominal distension, tenderness, low blood pressure and hemoglobin drop. Patient was re-explored for the second time, to identify the source of bleeding. There was an active bleeding in the hilum of left kidney due to renal artery perforation. Nephrectomy was performed. Renal specimen received in the pathology lab showed necrosis of renal parenchyma and necrotic material within the hilar vessels (Figure 2). Microscopic sections showed widespread coagulative necrosis containing fungal hyphea compatible with mucormycosis (Figure 3). Culture results of both the aspirated material and renal tissue showed mucorale and the genera mucor. Antifungal therapy was continued and after 10 days, he was discharged in a good health condition. Now after about 6 months, he is doing well and completely symptom-free.

## Discussion

Zygomycetes are opportunistic organisms with ubiquitous distribution in soil, decaying organic matter and air.[4] They have minimal pathogenicity but they got an aggressive behavior in immunosuppressant hosts.[1][2][5][6][7]

Mucormycosis which is alternately used as zygomycosis is caused by fungi of the order mucorale and the genera rhizopus, absidia and mucor.[4] Our case was diagnosed as the latter after culture. Four main presentations of the mucormycosis are rhinocerebral, pulmonary, gastrointestinal and disseminated forms.[2] Localized rhinocerebral involvement has been reported as the most common form in immunosuppressant hosts.[4]

Renal involvement of mucormycosis has been very rarely reported in the patients with different kinds of immunosuppressive conditions, but most the previously reported cases of renal mucormycosis are part of disseminated disease and isolated renal involvement is extremely rare.[3][4][7][8] To the best of our knowledge, less than 30 cases of isolated renal mucormycosis have been reported so far.[9] Most of the reported cases have been fatal and the diagnosis was made after autopsy, so antemortem diagnosis is very uncommon.[10]

Isolated renal mucormycosis has been reported in the patients with hematologic diseases such as aplastic anemia [4] and leukemia,[9] chronic obstructive pulmonary disease,[10] AIDS[6][7][11][12][13][14] diabetes mellitus[8] and organ transplant patients.[1][15][16] It has also been reported in the patients with no known underlying disease.[17][18][19]

In the transplant patients, all of the reported patients have been renal transplant and the proposed source of infection has been extrinsic via the transplanted organ.[1][15][16] In our case, the transplanted organ was liver from a deceased donor, so the definite source could not be identified. The three most common presenting symptoms are flank pain, hematuria and fever, which are mostly in favor of acute pyelonephritis.[4] However, the urine culture is sterile and the diagnosis cannot be made by urine culture.[8]

In our case, all of the cultures including surgical wound, blood and urine were negative.

Imaging studies has been claimed to be very useful in the organ transplanted patients.[6] Ultrasound shows an enlarged kidney with or without perinephric collection and CT scan, revealing an enlarged kidney with reduced or absent enhancement, and multiple low attenuation areas in the parenchyma.[4][5] All of the findings are in favor a renal abscess, which in the presence of sterile urine can be indicative of fungal infection.

Confirmation of the diagnosis needs tissue, either by fine needle aspiration or biopsy for histopathologic examination and culture.[5] In our patient, the first drained material was positive for fungus in favor of mucormycosis, which helped us for the definite diagnosis.

Successful therapy needs coordination of the surgeons and nephrologists, first by extensive debridement and amphotericin B which are the standard therapy.[6] However, according to the previous cases and also our experience; it seems that very few patients have survived without nephrectomy.[4][5][6] Our patient's fever continued despite of medical therapy and surgical intervention. At last, he developed active bleeding from renal artery because of arterial rupture secondary to angioinvasion of mucormycosis. The same problem has been previously reported in a combined kidney and liver transplantation.[1]

In conclusion, in an immunocompromised patient with flank pain and fever, as well as sterile urine and imaging studies in favor of abscess, fungal infection of the kidney and renal artery should be considered as a very important cause to prevent aggressive therapy and nephrectomy. Our case is the first isolated renal mucormycosis after liver transplantation that was successfully treated with nephrectomy.
